# The Degree of Hyperglycemia Excursion in Patients of Kidney Transplantation (KT) or Liver Transplantation (LT) Assessed by Continuous Glucose Monitoring (CGM): Pilot Study

**DOI:** 10.1155/2019/1757182

**Published:** 2019-11-27

**Authors:** Heung Yong Jin, Kyung Ae Lee, Yu Ji Kim, Tae Sun Park, Sik Lee, Sung Kwang Park, Hong Pil Hwang, Jae Do Yang, Sung-Woo Ahn, Hee Chul Yu

**Affiliations:** ^1^Division of Endocrinology and Metabolism, Department of Internal Medicine, Jeonbuk National University Medical School, Republic of Korea; ^2^Research Institute of Clinical Medicine of Jeonbuk National University- - Biomedical Research Institute of Jeonbuk National University Hospital, Jeonju, Republic of Korea; ^3^Division of Nephrology, Department of Internal Medicine, Jeonbuk National University Medical School, Republic of Korea; ^4^Division of Surgery, Jeonbuk National University Medical School, Republic of Korea

## Abstract

**Objective:**

This study used a continuous glucose monitoring system (CGMS) to investigate the glucose profiles and assess the degree of hyperglycemic excursion after kidney or liver transplantation during the early period after operation.

**Methods:**

Patients to whom a CGMS was attached during a postoperative period of approximately one month after transplantation were included. The CGM data of 31 patients including 24 with kidney transplantation (KT) and seven with liver transplantation (LT) were analyzed.

**Results:**

Hyperglycemia over 126 mg/dL (fasting) or 200 g/dL (postprandial) occurred in 42.1% (8/19) and 16.7% (1/6) of KT and LT patients, respectively, during this early period after transplantation, except for patients with preexisting diabetes (5 KT, 1 LT). The average mean amplitude of glycemic excursion (MAGE) and mean absolute glucose (MAG) levels were 91.18 ± 26.51 vs. 65.66 ± 22.55 (*P* < 0.05) and 24.62 ± 7.78 vs. 18.18 ± 7.07 (*P* < 0.05) in KT vs. LT patients, respectively, in patients without preexisting DM or PTDM patients who showed normal glucose levels. Average increase from the lowest level to the peak glucose value was higher in KT patients than LT patients (*P* < 0.05)*. Conclusions*. The transplanted organ also needs to be considered as an important factor affecting glucose control and the occurrence of more severe glucose excursions in patients who receive transplantation although immunosuppression agents are well-known important factors; however, our study was limited to the early posttransplantation period. Further studies involving CGM follow-up at regular intervals based on the time since transplantation are needed.

## 1. Introduction

Hyperglycemic excursion and difficulty of glucose control are well-known problems following solid organ transplantation in both nondiabetic patients and those with preexisting diabetes due to the administration of high or moderate doses of glucocorticoids and immunosuppressive agents [1, 2]. Among solid transplanted organs, worsened hyperglycemia is more common and severe in kidney transplantation (KT) patients because over 20% of KT patients suffer from end-stage renal disease (ESRD) caused by long durations of diabetes. Glucose control of preexisting diabetes can also be worsened by essential medications including glucocorticoids after transplantation [3]. Strict glucose control is very important in KT patients to prevent nephropathy in the transplanted kidney [4]. However, graft failure is also an important issue for patient survival following transplantation; therefore, immunosuppression is a more fundamental factor that should be kept in mind other than worsening of glucose control although transient or sustained hyperglycemia caused by these immunosuppressive agents or glucocorticoids or the development of posttransplantation diabetes mellitus (PTDM) can also negatively impact graft survival.

In addition to patients with KT, those with liver transplantation (LT) are also exposed to similar conditions after operation and there are no data to show the degree of hyperglycemia in comparison to that in KT. We previously reported that LT itself could be beneficial for glucose control in prediabetic and diabetic patients [5]. In this respect, glucose profiles after transplantation need to be differentiated according to the transplanted organ including organ-specific pathogenic mechanism as it is reported that glucose homeostasis can be deleterious due to beta cell dysfunction and inadequate insulin secretion in renal transplantation [6, 7].

During the early postoperative period, over 80% of transplant patients experience transient hyperglycemic episodes compatible to diabetic condition due to glucocorticoid use even though they had no history of diabetes. However, this persistent hyperglycemia cannot be determined to progress to PTDM at this time. The ideal time point to diagnose PTDM is an important issue in transplantation patients [1, 8, 9]. PTDM is not difficult to define with current diagnostic criteria; however, treatment timing and the decision to start an antihyperglycemic agent should be based on the glucose pattern and degree of hyperglycemia according to the time elapsed after transplantation. Therefore, the exact glucose tolerance state and detection of hyperglycemia over diabetes criteria compatible with PTDM are also important for glucose control, although this hyperglycemia may resolve over time with the tapering of glucocorticoid doses.

Continuous glucose monitoring (CGM) is well accepted as a technology that provides continuous and detailed information on glucose level trends, which can be used to precisely detect glucose fluctuation and guide patient treatment. Therefore, CGM can overcome the limitations of glycated hemoglobin (HbA1c) measures and self-monitoring blood glucose (SMBG) [4]. Therefore, the aim of this study was to investigate and compare the glycemic patterns and degree of glucose fluctuation during the early postoperative period after transplantation in KT and LT patients by using CGM data.

## 2. Methods

### 2.1. Patient Selection and CGMS Implantation

We conducted a retrospective observational study of 31 patients who underwent KT or LT at a single center of Jeonbuk National University Hospital in Jeonju, South Korea. All patients who underwent a KT or LT for the first time between September 2017 and March 2018 were included with approval of our hospital committee (institutional review board (IRB) approval No. 2018-12-017-001). The baseline patient demographics were collected before transplantation, and the medical records of the enrolled patients, as well as their CGM data after transplantation, were reviewed. The CGMS was implanted after stopping parenteral fluid therapy, and the patients had started oral diets similar to those of their routine lives before the operation. The CGMS were usually implanted about one week before discharge. Standard immunosuppression using tacrolimus and mycophenolate mofetil after transplantation was continued with glucocorticoid tapering over time.

### 2.2. Assessment of Glucose Profiles of Transplant Patients with CGMS and Comparison of Glycemic Variability according to Patient Groups

To detect hyperglycemia over the diabetic threshold and blood glucose profiles with fluctuation degree, 24-hour glucose monitoring was performed for 7 days using a wearable CGMS (iPro2, Medtronic Inc., USA). The sensor device was inserted under the skin on the upper arm, and interstitial glucose was checked every 5 minutes. The patients were instructed to calibrate the system with capillary blood glucose levels four times daily using a glucometer (Accu-Chek, Roche Diagnostics, USA). The CGMS was attached during the last week of hospitalization after transplantation to avoid the influence of parenteral nutrition and postoperative events. Hyperglycemia and hypoglycemia were defined as serum glucose levels > 200 and <70 mg/dL, respectively, in patients without preexisting diabetes. In addition to the degree of glucose fluctuation, the frequency of measurements over the 200 mg/dL threshold was important to detect overt hyperglycemia in patients without preexisting diabetes. The degree of glucose fluctuation was also assessed in patients with preexisting diabetes with glucose-lowering agents including insulin therapy. EasyGV Version 9.0.R2 was used to calculate the variables of glycemic variability (http://www.easygv.co.uk/), as described previously [10, 11]. To precisely measure glycemic patterns in detail, the following indices of glucose control quality and variability were calculated in addition to the mean and SD values: weighted average glucose values (M-VALUE), high blood glucose index (HBGI), average daily risk range (ADRR), combination of information from mean and SD of all glucose values (J-INDEX), continuous overlapping net glycemic action (CONGA), lability index (LI), mean of daily differences (MODD), mean amplitude of glycemic excursion (MAGE), and mean absolute glucose (MAG). These parameters were calculated and interpreted as described previously [12]. In addition, the glucose profiles of patients without preexisting diabetes before transplantation were analyzed to detect PTDM. Hyperglycemia (glucose levels over 200 mg/dL and 126 mg/dL before breakfast) was considered as the threshold for PTDM according to the seven-day CGM record. To assess the effect of glucocorticoids on glucose fluctuation, we also compared the average glucose levels for the time periods of 3-6 AM, 6-9 AM, 12-15 PM, and 15-18 PM in all KT and LT patients.

### 2.3. Statistical Analysis

All data are expressed as means ± standard deviation (SD). Continuous variables were compared using *t*-tests. Statistical significance was accepted for *P* values < 0.05 with 95% confidence intervals. Statistical analysis was performed using PASW Statistics for Windows, version 18.0 (SPSS Inc., Chicago, IL, USA).

## 3. Results

### 3.1. Baseline Characteristics of KT and LT Patients

Our analysis included 31 patients who underwent KT (*n* = 24) or LT (*n* = 7). Five patients in the KT group and one patient in the LT group had preexisting DM before transplantation. The mean ages were 51.2 ± 8.9 and 46.7 ± 9.0 years, and there were 16/8 and 3/4 of men and women in the KT and LT groups, respectively (Table 1). At one month after transplantation, the patients (excluding those with preexisting diabetes) were categorized as euglycemia and hyperglycemia according to the CGM data and baseline characteristics, as summarized in Table 2.

### 3.2. CGM Patterns of KT and LT Patients according to the Presence of Preexisting Diabetes and Postoperative Hyperglycemia

**Figure 1:**
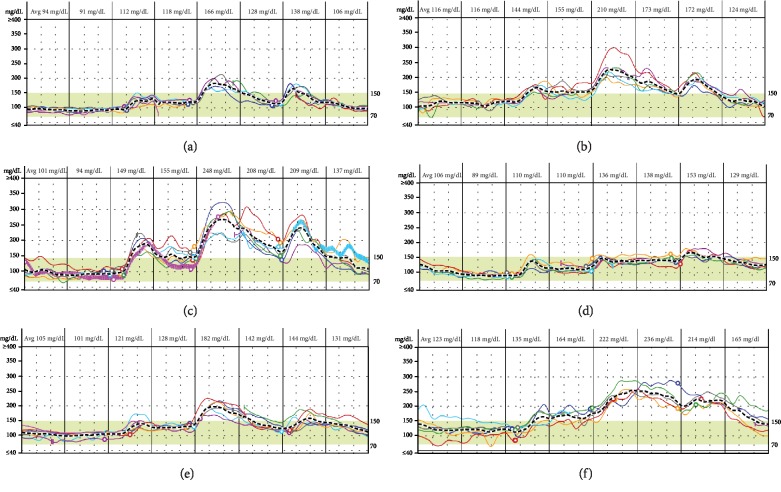
CGM patterns during seven days of the early period after operation according to the presence of diabetes in KT and LT patients. (a) CGM in a KT patient without PTDM and without preexisting diabetes. (b) CGM in a KT patient with PTDM without preexisting diabetes. (c) CGM in a KT patient with preexisting diabetes. (d) CGM in an LT patient without PTDM and without preexisting diabetes. (e) CGM in an LT patient with PTDM and without preexisting diabetes. (f) CGM in an LT patient with preexisting diabetes. KT: kidney transplantation; LT: liver transplantation; PTDM: posttransplantation diabetes mellitus.

The glucose profiles after transplantation varied according to the patient's glucose tolerance state; however, most patients showed different patterns of glucose fluctuation according to group. In euglycemia patients after KT or LT, fasting and random glucose levels were maintained below the normal glucose threshold. However, hyperglycemia patients without preexisting DM showed glucose levels above the diagnostic glucose value for diabetes based on the American Diabetes Association (ADA) criteria [13]. Although patients with preexisting DM did not show consistent glucose patterns, the fluctuations observed in their glucose levels were severe compared to those observed in the other groups (Figures 1(a)–1(f)).

### 3.3. Comparison of PTDM Occurrence between KT and LT Groups during the Early Postoperative Period Based on CGM Data

**Figure 2:**
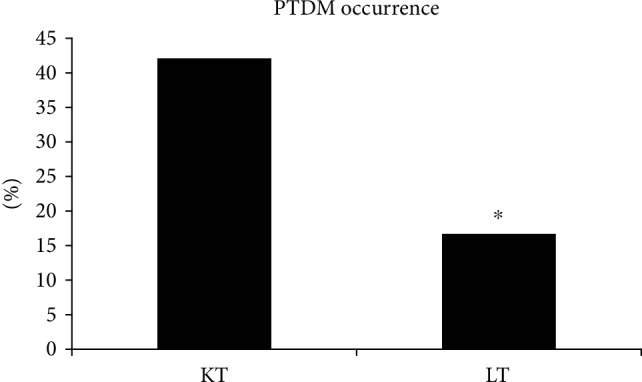
Comparison of PTDM occurrence between KT and LT patients based on CGM data. More KT than LT patients showed a hyperglycemic (glucose over 126 mg/dL at fasting or 200 mg/dL at postprandial 2 hr point) state. KT: kidney transplantation; LT: liver transplantation; PTDM: posttransplantation diabetes mellitus. Values are presented as percentages.

The patient compatible to PTDM in respect of fasting glucose or postprandial glucose value was more common in the KT group than in the LT group, as shown in Figure 2, although the number of LT patients was small and LT patients were not matched to the KT group in baseline characteristics. Among the 19 patients in the KT group, eight had hyperglycemia over the diabetic fasting glucose threshold of 126 mg/dL or postprandial glucose threshold of 200 mg/dL. Among the six patients in the LT group, only one had CGM data over these thresholds.

### 3.4. Comparisons of Glycemic Variability between KT and LT Patients during the Early Postoperative Period

**Figure 3:**
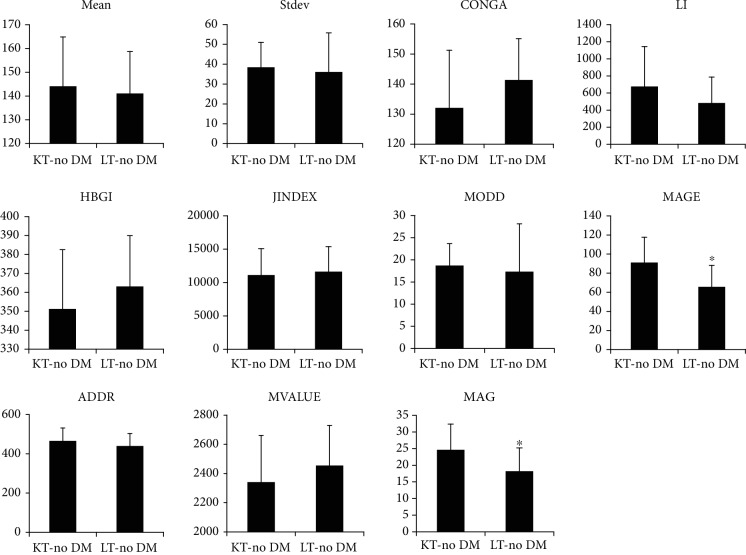
Comparison of diverse indices representing glycemic variability between the KT and LT groups excluding patients with preexisting DM and PTDM. In patients without DM, a similar degree of glucose fluctuation was observed for all indices of glycemic variability except for MAGE and MAG, which showed significantly less fluctuation in the LT group than in the KT group. Data are presented as the mean ± SD. ^∗^Significant difference (*P* < 0.05) between the KT and LT groups.

**Figure 4:**
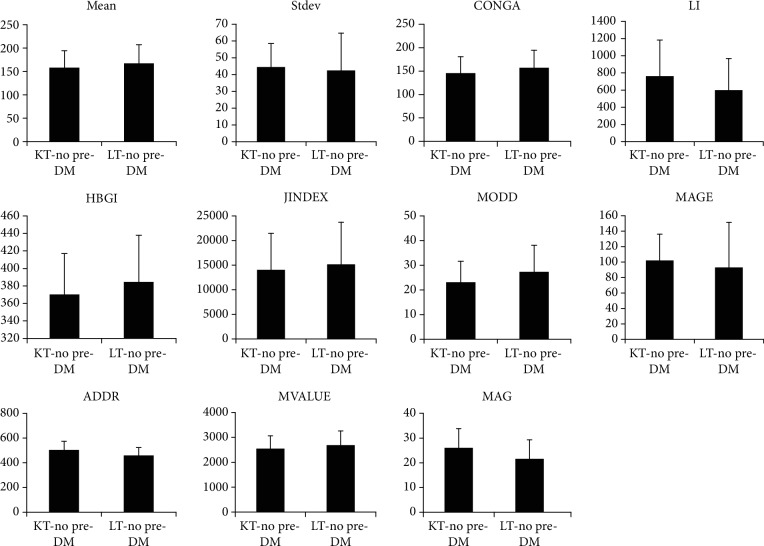
Comparisons of various indices representing glycemic variability between the KT and LT groups excluding only preexisting DM patients. In patients with normal glucose values or PTDM, there were no significant differences between the KT and LT groups for all indices. Data are presented as the mean ± SD.

As summarized in Table 3, the various parameters of glucose fluctuation did not differ significantly between the KT and LT groups; however, patients with preexisting DM were included in these comparisons. When preexisting DM and PTDM patients were excluded and only patients who showed normal glucose values were compared, those in the LT group had significantly lower MAGE and MAG values than those in the KT group. There were no significant differences in the other indices. The status of glycemic control and glycemic variability in nondiabetic patients after LT were better than those of KT patients, as indicated by MAGE and MAG as follows (mean 144.08 ± 20.83 vs. 141.02 ± 17.73, *P* > 0.05; SD 38.49 ± 12.55 vs. 36.11 ± 19.68, *P* > 0.05; CONGA 132.07 ± 19.18 vs. 141.38 ± 13.72, *P* > 0.05; J-INDEX 11,121.38 ± 3,960.25 vs. 11,620.37 ± 3,762.50, *P* > 0.05; HBGI 351.23 ± 31.29 vs. 363.11 ± 26.88, *P* > 0.05; MODD 18.72 ± 4.93 vs. 17.33 ± 10.81, *P* > 0.05; MAGE 91.18 ± 26.51 vs. 65.66 ± 22.55, *P* < 0.05; ADDR 465.04 ± 65.91 vs. 439.30 ± 63.55, *P* > 0.05; M-VALUE 2,340.57 ± 320.17 vs. 2,455.45 ± 274.06, *P* > 0.05; and MAG 24.62 ± 7.78 vs. 18.18 ± 7.07, *P* < 0.05, respectively). These indices are shown in Figure 3. In patients without only preexisting DM, there were also no significant differences between the KT and LT groups in all indices of glycemic variability, as shown in Figure 4. However, patients with only PTDM could not be compared because there was only one patient who was considered as PTDM in the LT group.

### 3.5. Degrees of Glucose Excursion according to the Time of Day in the KT and LT Groups

**Figure 5:**
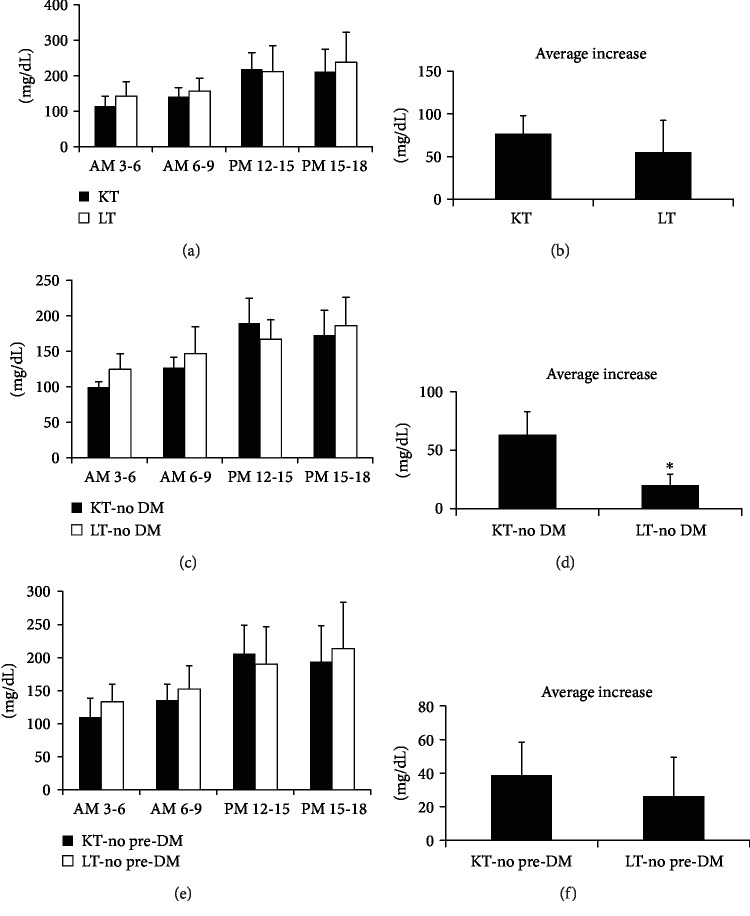
Comparison of the average glucose excursion according to the time interval in a day in the KT and LT groups according to the presence of preexisting DM or PTDM. (a, b) Both preexisting DM and PTDM patients and (c, d) both preexisting DM and PTDM patients, and only preexisting DM patients were excluded in (e, f). (a) Glucose levels increased trend in the afternoon and early evening intervals in both KT and LT groups which included both preexisting DM and PTDM; however, there were no significant differences in the glucose levels between the KT and LT groups during all time intervals. (b) The average increase in the glucose level tended to be lower in the LT group than that in the KT group; however, the difference was not statistically significant. (c) After excluding patients with preexisting diabetes and PTDM, both KT and LT groups with a normal glucose state showed similar increasing trends in glucose levels in the afternoon and early evening time intervals; however, the differences were also not significant in every time interval. (d) The average increase in the glucose level was significantly lower in the LT group than that in the KT group after excluding patients with preexisting DM and PTDM. (e) KT and LT patients after excluding only preexisting DM showed a similar trend to those of the other comparison. (f) The average increase in the glucose level between the KT and LT groups without preexisting DM did not show significant difference. Data are presented as the mean ± SD. ^∗^Significant difference (*P* < 0.05) between KT and LT.

In both KT and LT groups, the average glucose level showed an increasing trend during the afternoon and early evening time points compared to the level during the morning periods regardless of diet. There were no significant differences in glucose levels within the same time intervals between the KT and LT groups (Figures 5(a), 5(c), and 5(e)); however, after excluding both preexisting DM and PTDM, the average glucose increase during the afternoon interval compared to the fasting level was lower in the LT group than that in the KT group in patients with a normal glucose state (*P* < 0.05) (Figure 5(d)). The increase in the average glucose values showed similar trends in the KT and LT patients when both preexisting DM and PTDM patients were included or PTDM patients were included (Figures 5(b) and 5(f)). A few patients experienced hypoglycemia with glucose levels below 70 mg/dL during the monitoring periods in both KT and LT patients except with the use of glucose-lowering agents including insulin in patients with preexisting DM.

## 4. Discussion

Our study used the indices of glycemic variability of CGMS to assess and try to compare the detailed patterns of glucose fluctuation between KT and LT patients during the early period after transplantation. In addition, we investigated the potential differences in the degree of fluctuation among transplantation patients according to the transplanted organ. We found a pattern of reduced PTDM occurrence and less severe glycemic fluctuations in LT patients than in KT patients although the differences in glycemic variability were based on a limited number of indices such as MAGE and MAG in patients with euglycemia patients. Our study also confirmed previous findings that the hyperglycemia pattern after transplantation showed increased levels in the afternoon and early evening time periods due to the administration of glucocorticoids in the morning. Our study also showed the detailed degree of the increase in glucose levels, although a long-term follow-up over 6 months or at least 1 year is necessary to support our results. Previously, this pattern after KT was reported using CGM [11]; however, to our knowledge, no previous report has compared the glycemic profiles of KT and LT patients.

Hyperglycemia may occur after transplantation in patients without a medical history of diabetes, and transplantation may also worsen hyperglycemia in patients with preexisting DM. The pattern and severity of hyperglycemia in nondiabetic patients may differ from those in patients with preexisting DM. Patients undergoing transplantation may be exposed to several issues, including treatment with immunosuppressive agents such as glucocorticoids [11, 14, 15]. Therefore, in the early posttransplantation period, either glucocorticoid-induced hyperglycemia may occur or poor glucose control including severe glucose fluctuation is common, and the severity of which also depends on the preexisting glucose tolerance state. However, there is little information on glucose patterns and fluctuation degree that occur after transplantation in nondiabetic or diabetic patients. Clarification of these profiles is helpful to document PTDM and control glucose levels meticulously in patients with preexisting DM. According to their pretransplantation glucose status, patients can be categorized as follows: nondiabetic patients who showed hyperglycemia compatible to PTDM or who maintain normal glucose tolerance and diabetic patients who experience worsening of their glucose control or who benefit from the transplanted organ for glucose control. Of course, it is important to assess the glucose state with respect to the time duration since transplantation to confirm PTDM. Posttransplantation patients who developed hyperglycemia in the immediate period following the operation should be categorized into two groups depending on the presence or absence of preexisting DM [1]. In transplantation patients, CGM is helpful to detect hyperglycemia that could not be diagnosed as PTDM by HbA1c or fasting glucose value [16]. Moreover, HbA1c or 75 g OGTT is inadequate during the early period after operation. Patients with preexisting DM showed exacerbated glucose fluctuation with overt hyperglycemia, and more intense strategies were required, especially during the postoperative period. Therefore, CGM can be used to guide glucose control in patients with preexisting DM as well to detect diabetes in nondiabetic patients during the posttransplantation period. Therefore, CGM may be helpful for the accurate diagnosis of diabetes and documentation of the detailed glucose control state in this period although PTDM needs to be identified according to the presence of sustained hyperglycemia in transplantation patients without preexisting DM. Moreover, prognostic superiority of CGM in comparison to the frequent bedside glucose check or self-glucose monitoring in the early posttransplant period can be suggested as follows. First, more precise detection for abnormal glucose levels is possible by using CGM, and it is better to control glucose levels strictly than self-glucose monitoring. Good glycemic control should be maintained for improvement of graft survival. Second, PTDM occurrence can be more carefully monitored in the follow-up of transplantation patients based on the CGM data. Transplantation patients showing intermittent glucose fluctuations over the diabetes threshold that cannot be detected by self-glucose monitoring need to be carefully monitored whether PTDM would occur or not more thoroughly compared with patients showing normal glucose range in the data of CGM.

Among diverse organ transplantations, KT and LT are closely related to the glucose level in two ways. The transplanted organ itself can influence glucose homeostasis because healthy transplanted liver or kidneys may play a positive role in glucose control in both nondiabetic and diabetic patients. However, recipients of transplantation require long-term immunosuppression, which can negatively impact glucose control owing to the use of immunosuppressive agents that increase glucose levels. Therefore, glycemic levels may either be normalized or become unbalanced to the extent of developing PTDM following KT or LT. However, the mechanism underlying such developments is unclear, and there are very few reports on such glycemic changes over time following KT and LT, although Aouad et al. used CGM to assess changes in glucose levels after KT [11]. Moreover, the measurement of glycemic variability by CGM in diabetic patients is widely accepted for the detection of hypoglycemia and precise assessment of the treatment state, and micro- and macrovascular complications have been postulated as being partly related with glycemic variability [17, 18]. Glucose levels peaked in the afternoon in both KT and LT patients, as reported by previous studies [11, 19]. In our study, no significant differences were observed between KT and LT patients, and the average increase was lower in LT patients irrespective of the higher dose of glucocorticoids in the LT group. Although this might be attributable to the individual susceptibility and dose of glucocorticoids administered, LT patients may have a less severe peak in glucocorticoid-induced glucose fluctuation than KT patients.

PTDM after KT occurs in 10%-40% of patients without preexisting DM [7, 20]. Postoperative stress, immunosuppressive agents, and high doses of glucocorticoids have metabolic effects that can cause glucose elevation or worsen hyperglycemia in patients with and without preexisting DM [7, 21, 22]. Postoperative inpatient hyperglycemia after KT has been recently reported [1]. The early and exact identification of hyperglycemia in transplantation patients during the immediate postoperative period is important not only to monitor and control hyperglycemia but also to predict PTDM and reduce adverse outcomes including those pertaining to graft survival in transplantation [23]. Regardless of the fact that LT patients received a higher dose of glucocorticoids than KT patients during this period, LT patients showed a lower incidence of hyperglycemia and blunted glucose fluctuation in comparison with KT patients in our study. Therefore, in addition to baseline patient characteristics and glucocorticoid dose, different glucose responses among transplantation types should be considered based on the transplanted organ.

Several factors can influence the glucose levels in organ transplantation patients. Generally, these patients are managed with immunosuppressive agents including glucocorticoids, which may have negative effects on glucose homeostasis in both patients with preexisting DM and those without DM. However, the potential role of the transplanted organ in glucose control should also be considered. As beneficial effect of the healthy transplanted liver on glucose homeostasis due to the positive effects on the control of gluconeogenesis and hepatic insulin resistance in patients with and without preexisting DM [5], the effect of a healthy kidney on the glucose control should also be considered in KT patients. However, there are no data on the potential beneficial role of the transplanted kidney in the direct or indirect mechanism for glucose control in KT patients. Of course, our data also showed a similar pattern of hyperglycemia to those in previous studies, with an increase from midmorning to afternoon until early evening due to morning glucocorticoid administration [24, 25].

In addition to the fasting plasma glucose (FPG) level, the HbA1c level and 75 g OGTT findings can be used to detect diabetes in the normal population. These parameters are also useful in transplantation patients for the diagnosis of PTDM; however, these values may not reflect the exact glucose status during the early period following the operation, and the exact glucose state is difficult to confirm in these patients due to parenteral nutrition therapy and diverse postoperational factors. Moreover, exact glucose profiles in organ transplantation patients are required for glucose control because preexisting DM patients are also exposed to this situation and exact detection of PTDM is an important issue for transplantation patients. Our study did show the glucose control state exactly during the postoperative period after transplantation and a little difference in the glucose pattern between KT and LT patients.

Clinical implications of our study can be summarized as follows. First, this study helps clinicians predict the pattern and degree of glucose fluctuation in the care of transplantation patients. Therefore, it will be helpful to determine the dose or pharmaceutical class of hypoglycemic agents. Second, CGM may be useful to detect PTDM more exactly than HbA1c or 75 g OGTT in the early period of posttransplantation and can be used as a tool to assess the risk of PTDM development during the follow-up of transplantation patients.

However, our study has several limitations. First, the patients in our study may have included those with transient hyperglycemia due to glucocorticoid treatment, and they may be categorized as PTDM patients although their glycemic levels might normalize after discontinuation of glucocorticoid treatment. Therefore, long-term follow-ups at regular intervals are necessary to confirm whether PTDM is sustained in addition to monitoring the changes of glucose fluctuation. The comparison of the posttransplant 6-month and/or 12-month glucose status including glucose homeostasis, insulin resistance, and PTDM occurrence is also helpful to support the importance of these high MAGE and MAG meaning in their early posttransplant period in the care of transplantation patients. Second, insulin and medication changes after transplantation in the preexisting DM group are needed to be analyzed to note the actual effects of the transplanted organs on glucose control, but we did not perform this comparison because of the small number of patients in these groups. Third, after the withdrawal of glucocorticoid treatment, comparison of 75 g OGTT and HbA1c and follow-up with CGM data is helpful for the exact diagnosis of PTDM. Fourth, we could not determine differences in the change of medication or insulin dose in preexisting DM and PTDM patients in the KT and LT groups because of the small number of patients. Of course, the study population is very small to conclude any solid data about the glucose excursion patterns at this point especially in liver transplant patients. Therefore, more CGM data measured from LT patients are warranted in the future.

Conclusively, one novel aspect of our study was to assess and compare the degree of glucose fluctuation between KT and LT patients by CGM. The glucose fluctuation patterns, as measured by the MAGE and MAG, were less severe after LT compared to those after KT in patients with normal glucose status. Moreover, the occurrence of hyperglycemia considered to be PTDM was lower in LT than that in KT patients. Therefore, further research is necessary to investigate whether LT is more beneficial than KT with respect to glycemic variability and glucose control in PTDM or transplant patients with preexisting DM. Moreover, diverse studies with CGM in transplant patients are warranted for the exact diagnosis of diabetes and optimized glucose control in both patients with preexisting DM and those who develop PTDM.

## Figures and Tables

**Table 1:** Baseline characteristics of all kidney or liver transplantation patients.

	KT patients	LT patients
Male/female	24 (16/8)	7 (3/4)
Age (yr)	51.2 ± 8.9	46.7 ± 9.0
Body weight (kg)	67.7 ± 12.0	58.0 ± 6.8
Height (cm)	165.5 ± 8.65	161.7 ± 5.68
BMI (kg/m^2^)	24.5 ± 3.26	22.1 ± 1.60
Prednisolone (mg)	10	30
Tacrolimus (mg)	4.0	8.0
FBS (mg/dL)	121.2 ± 47.0	129.3 ± 59.51
PPS2hr (mg/dL)	209.9 ± 57.1	215.2 ± 85.06
HbA1c (%)	5.26 ± 0.7	6.02 ± 2.48
Insulin (*μ*u/mL)	6.4 ± 3.3	7.16 ± 4.15
C-peptide (ng/mL)	3.65 ± 1.20	3.56 ± 1.5
T.cholesterol (mg/dL)	180.0 ± 33.1	155.2 ± 30.65
TG (mg/dL)	126.0 ± 54.1	172.8 ± 69.3
HDL (mg/dL)	55.1 ± 23.2	53.8 ± 14.7
LDL (mg/dL)	90.2 ± 21.8	77.2 ± 32.1

**Table 2:** Baseline characteristic of patients excluding preexisting diabetes in each KT and LT groups.

	KT without preexisting DM		LT without preexisting DM	
	Non-PTDM	PTDM	Non-PTDM	PTDM
Male/female	7/4	5/3	3/2	1/0
Age (yr)	47.9 ± 9.27	53.3 ± 9.75	45.5 ± 10.27	55
Body weight (kg)	70.9 ± 15.89	63.9 ± 8.64	57.8 ± 8.67	60.1
Height (cm)	165.2 ± 10.97	164.1 ± 5.56	161.5 ± 7.27	163.2
BMI (kg/m^2^)	25.5 ± 3.60	23.7 ± 2.45	22.1 ± 2.0	22.56
Prednisolone (mg)	10	10	30	30
Tacrolimus (mg)	4	4	8	8
FBS (mg/dL)	88.8 ± 16.22	117.9 ± 32.66	84.8 ± 13.93	112 ± 26.8
PPS2hr (mg/dL)	131.1 ± 63.05	185.8 ± 40.31	124.3 ± 33.53	170 ± 35.5
HbA1c (%)	4.89 ± 0.62	5.18 ± 0.44	4.6 ± 0.33	4.3 ± 0.42
Insulin (*μ*u/mL)	6.2 ± 1.9	6.6 ± 2.4	7.16 ± 2.15	7.5 ± 2.5
C-peptide (ng/mL)	3.4 ± 0.9	3.9 ± 1.2	3.26 ± 1.2	3.78 ± 1.8
T.cholesterol (mg/dL)	180.5 ± 35.20	181.9 ± 20.46	145.5 ± 29.22	±152
TG (mg/dL)	132.6 ± 58.98	129.1 ± 62.17	112.5 ± 41.07	76
HDL (mg/dL)	53.6 ± 20.96	59.5 ± 20.46	50.8 ± 16.72	±68
LDL (mg/dL)	90.7 ± 19.94	91.6 ± 27.53	70 ± 30.07	71

**Table 3:** The comparison of indices of glycemic variability between all KT and LT patients including preexisting DM.

	KT	LT
Mean	158.1 ± 36.30	186.8 ± 59.37
SD	44.5 ± 14.40	48.7 ± 25.0
CONCA	145.8 ± 35.90	176.6 ± 58.06
LI	763.4 ± 431.25	655.9 ± 356.04
JINDEX	14,038.3 ± 7,649.31	19,779.5 ± 13,660.42
HBGI	370.3 ± 47.90	408.0 ± 74.70
MODD	23.1 ± 8.54	27.3 ± 10.80
MAGE	102.2 ± 33.95	113.0 ± 71.46
ADDR	504.0 ± 69.98	459.3 ± 63.55
M-VALUE	2,543.4 ± 515.26	2,949.5 ± 819.72
MAG	26.1 ± 7.75	22.8 ± 7.45

Data are presented as the mean ± SD. ^∗^Significant difference (*P* < 0.05) between KT and LT.

## Data Availability

The data used to support the findings of this study are available from the corresponding or 1st author upon request.
